# Claudin-4 controls the receptor tyrosine kinase EphA2 pro-oncogenic switch through β-catenin

**DOI:** 10.1186/s12964-014-0059-5

**Published:** 2014-10-25

**Authors:** Xiying Shang, Xinjian Lin, Stephen B Howell

**Affiliations:** Department of Medicine and the Moores UCSD Cancer Center, University of California, 3855 Health Sciences Drive, La Jolla, San Diego, CA 92093-0819 USA

## Abstract

**Background:**

The EphA2 receptor, which is expressed in many types of cancer, is activated by two different mechanisms. Activation by engagement with one of its ephrin ligands is anti-oncogenic whereas phosphorylation of S897 by AKT increases migration, invasion and metastasis. Down-regulation of claudin-4 (CLDN4) produces a loss of E-cadherin and increased β-catenin signaling and a phenotype similar to that produced by oncogenic activation of EphA2, suggesting that CLDN4 may serve to restrain the pro-oncogenic signaling of EphA2.

**Results:**

We found that constitutive knockdown of CLDN4 was associated with a 4.5-fold increase in EphA2 mRNA and a 2.5-fold increase in EphA2 protein which was reversible by re-expression of CLDN4. Knockdown of EphA2 blocked the migratory phenotype induced by loss of CLDN4. Knockdown of CLDN4 resulted in a 5.8-fold increase in pEphA(S897), the oncogenic form of the receptor, as well as partial mislocalization of the excess EphA2 to the interior of the cell. Forced expression of E-cadherin did not reduce total EphA2 or pEphA(S897) whereas re-expression of CLDN4 restored localization and reduced EphA2 and pEphA(S897) even in cells not expressing E-cadherin. Transient siRNA-mediated knockdown of EphA2 and β-catenin, and inhibition of PI3K by LY294002, demonstrated that increased pEphA(S897) in the CLDN4 knockdown cells was attributable to an increase in the level of active dephospho-β-catenin upstream of PI3K and AKT.

**Conclusions:**

We conclude that CLDN4 serves to restrain pro-oncogenic signaling from EphA2 by limiting the activity of β-catenin and PI3K and preventing phosphorylation of EphA2 on S897 by AKT. This suggests that interventions directed at enhancing the level or functional activity of CLDN4 may be of therapeutic interest.

**Electronic supplementary material:**

The online version of this article (doi:10.1186/s12964-014-0059-5) contains supplementary material, which is available to authorized users.

## Background

Eph receptors make up the largest family of receptor tyrosine kinases (RTK). There are 14 distinct Eph receptors and they interact with 8 membrane-bound ligands known as ephrins. There are two subfamilies of Eph receptors, EphA and EphB. The 9 EphA receptors expressed in humans bind 5 different glycosyl phosphatidylinositol (GPI)-linked ephrin-A ligands and the 5 EphB receptors bind 3 transmembrane ephrin-B ligands [1]. EphA2 is of particular interest because it is up-regulated in many tumors and its expression frequently correlates with an aggressive phenotype [2-7]. One of the unique features of the Eph/ephrin interaction is that it generates signals that propagate in both directions; by convention the signaling activated in the Eph-expressing cell is considered forward signaling.

EphA2 can be activated by two different mechanisms. Activation by interaction with ephrin-A1 causes phosphorylation of EphA2 that generates an anti-oncogenic signal as shown by the observation that forced activation by exposure to soluble ephrin-A1 can inhibit tumor growth both *in vitro* and *in vivo* [8,9]. Although ephrinA1 is expressed at low levels in cancer cells it can robustly activate EphA2 upon release into the extracellular environment [10]. Activation by the ephrin-independent pathway involves activation of PI3K and phosphorylation of AKT which in turn phosphorylates EphA2 on serine 897 in the cytoplasmic tail [11]. Increasing the phosphorylation of EphA2 in this manner generates an oncogenic signal that results in increased EphA2-dependent cell migration and invasion [9,11]. Thus, the signals generated by EphA2 can be switched from anti-oncogenic to oncogenic direction depending on how this kinase is phosphorylated, and the net effect is the result of balance between the two activation mechanisms. Despite its frequent over-expression on malignant cells, when tumors are grown *in vivo* EphA2 appears to be only poorly activated by the ephrin-A1 anchored on the membrane of adjacent cells and thus the output in this setting is largely oncogenic.

One of the hallmarks of malignant epithelial cells is that the cell-cell junctions, particularly the tight junctions (TJ), are disassembled, remodeled or lost [12-17]. We reported previously that knockdown of claudin-4 (CLDN4) in a human cervical cancer cell line 2008 markedly increases the growth of its xenografts and enhances their metastatic potential through down-regulation of E-cadherin mRNA and protein levels [18]. Interestingly, it has been demonstrated that E-cadherin promotes EphA2–ephrin-A1 interaction at the cell–cell junctions by stabilizing intercellular contacts, which in turn enhance the EphA2 forward anti-oncogenic signaling [9].

We report here that CLDN4 can control the switch from the ephrin-A1-dependent anti-oncogenic to the AKT-dependent pro-oncogenic signaling by decreasing the phosphorylation of EphA2 at S897 through inhibition of active β-catenin. Constitutive knockdown of CLDN4 in the 2008 cells increased the amount of active β-catenin and the pAKT(S473) and pEphA2(S897) levels which in turn drive the AKT-EphA2 pro-oncogenic signaling cascade in a ligand independent manner. Re-expression of CLDN4, or forced expression of CLDN4 in cells in which it is not endogenously expressed, was found to restrain AKT-EphA2 pro-oncogenic signaling.

## Results

### CLDN4 knockdown modulates the expression of EphA2

**Figure 1:**
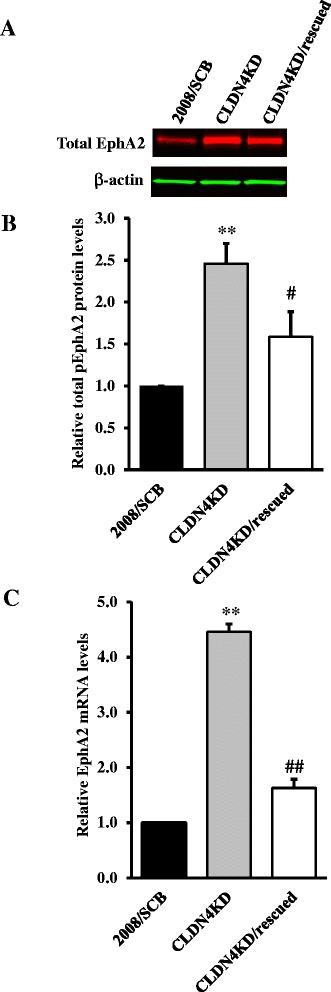
**Effect of CLDN4 on EphA2 expression. (A)** Representative Western blot showing that CLDN4 knockdown increased EphA2 level, and that this was partially reversed by re-expression of CLDN4 expression. **(B)** The histogram shows the mean level of EphA2 protein determined from 3 independent experiments expressed as the fold change relative to that in the scrambled siRNA control 2008/SCB cells after normalization to β-actin. **(C)** qRT-PCR analysis showing that knockdown of CLDN4 increased EphA2 mRNA level and that this was partially reversed by re-expression of CLDN4. Results are given as mean ± SEM (n = 3–4). ^**^ *p* < 0.01 versus 2008/SCB; ^##^ *p* < 0.01 versus CLDN4KD.

The over-expression of EphA2 found in many aggressive tumors is associated with increased proliferation and migration [19,20], and this has been linked to ligand-independent activation of EphA2 through phosphorylation on S897 [11]. In a previous study [18] we molecularly engineered the cervical carcinoma 2008 cell line to create a CLDN4KD subline in which the expression of CLDN4 was constitutively knocked down as a result of infection with a lentivirus expressing an shRNAi targeted to the CLDN4 mRNA. The level of both CLDN4 mRNA and protein was decreased by 80-90% compared with the parental 2008 cells infected with the control virus expressing a scrambled shRNAi which did not target any human gene, designated here as 2008/SCB cells [18]. Western blot analysis was used to determine whether the aggressive phenotype of the CLDN4KD cells was linked to changes in the expression of EphA2. As shown in Figure 1A and 1B, knockdown of CLDN4 resulted in a 2.5 ± 0.1-fold increase in total EphA2 protein (*p* < 0.01). To document the specificity of this effect, CLDN4 was stably re-expressed in the CLDN4KD cells by infecting them with a lentivirus coding for a CLDN4 mRNA containing 6 mutations that did not alter the amino acid sequence but abrogated recognition by the shRNAi to create the CLDN4KD/rescued cells. Re-expression of CLDN4 partially reversed the effect of CLDN4 knockdown and reduced total EphA2 by 35.7% (*p* > 0.05) to a level of 1.6 ± 0.2 -fold above that in the control 2008/SCB cells. The change in total EphA2 induced by knockdown of CLDN4 was accompanied by an even larger change in EphA2 mRNA as quantified by qRT-PCR. Figure 1C shows that CLDN4 knockdown increased EphA2 mRNA by 4.5 ± 0.14 -fold (*p* < 0.01), and that re-expression of CLDN4 partially reduced this by 63.5% (*p* < 0.01) to the extent of 1.6 ± 0.16 -fold above that in the 2008/SCB cells. Thus, knockdown of CLDN4 produced substantial increases in both EphA2 mRNA and protein levels.

### EphA2 signaling is responsible for the enhanced migration observed in CLDN4KD cells

**Figure 2:**
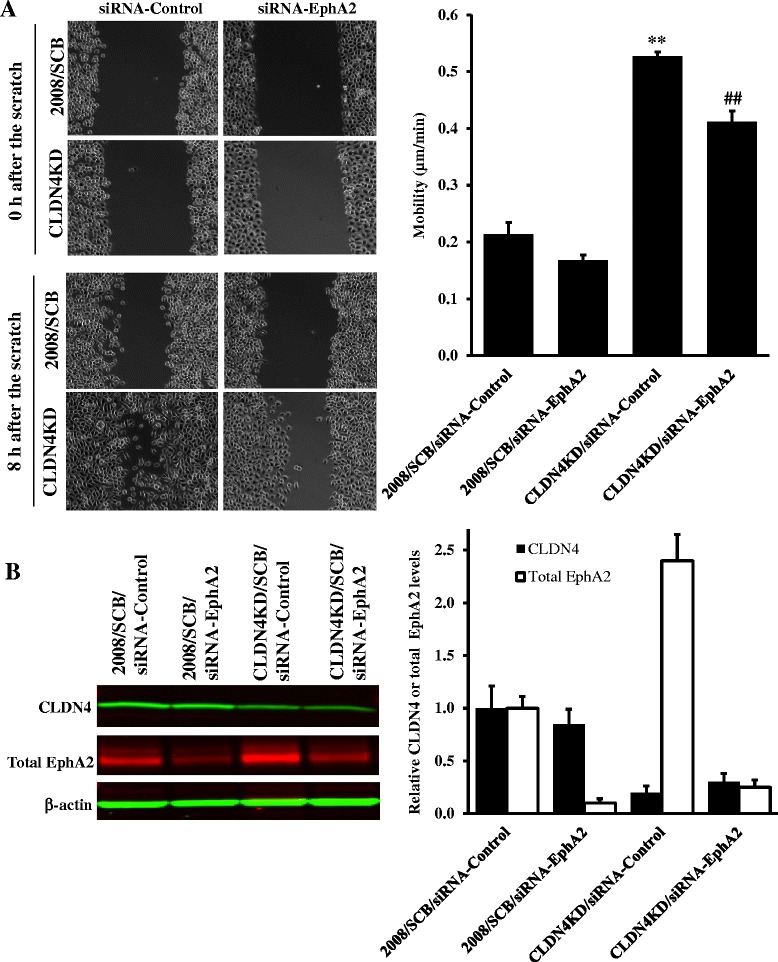
**Effect of siRNA-mediated knockdown of EphA2 on migration of 2008/SCB and CLDN4KD cells. (A)** Relative motility was determined by the ability of 2008/SCB and CLDN4KD cells with or without knockdown of EphA2 expression to close a wound made by creating a scratch through a lawn of confluent cells. Cell images were taken at 0 and 8 h after the scratch. The histogram shows quantification of the migration speed from two independent experiments each performed with triplicate cultures. Migration distance (μm) travelled 8 h after the scratch was determined using Slidebook (v5.0) software and average motile speed (μm/min) was calculated. Values are the mean ± SEM; ^**^ *p* < 0.01 versus 2008/SCB/siRNA-Control, ^##^ *p* < 0.01 versus CLDN4KD/siRNA-Control. **(B)** Expression of CLDN4 and EphA2 in the indicated four cell lines by Western blot analysis. The histogram summarizes the results of 3 independent experiments expressed as the fold change relative to that in the scrambled siRNA control 2008/SCB cells after normalization to β-actin. Results are reported as mean ± SEM (n = 3).

We previously reported that knockdown of CLDN4 increased the migration, invasion and metastatic potential of 2008 cells [18]. To determine the extent to which the enhanced migration was mediated by EphA2, the 2008/SCB and CLDN4KD cells were treated with scrambled siRNA or siRNA targeted to EphA2 which reduced the level of EphA2 protein by >50%. As shown in Figure 2, knockdown of CLDN4 significantly increased the migration rate whereas concomitant knockdown of EphA2 partially reversed this effect despite the fact that the knockdown of EphA2 was not complete. Thus, the migratory behavior of the CLDN4KD cells was modulated by the level of EphA2.

### CLDN4 knockdown alters the phosphorylation and kinase activity of EphA2

**Figure 3:**
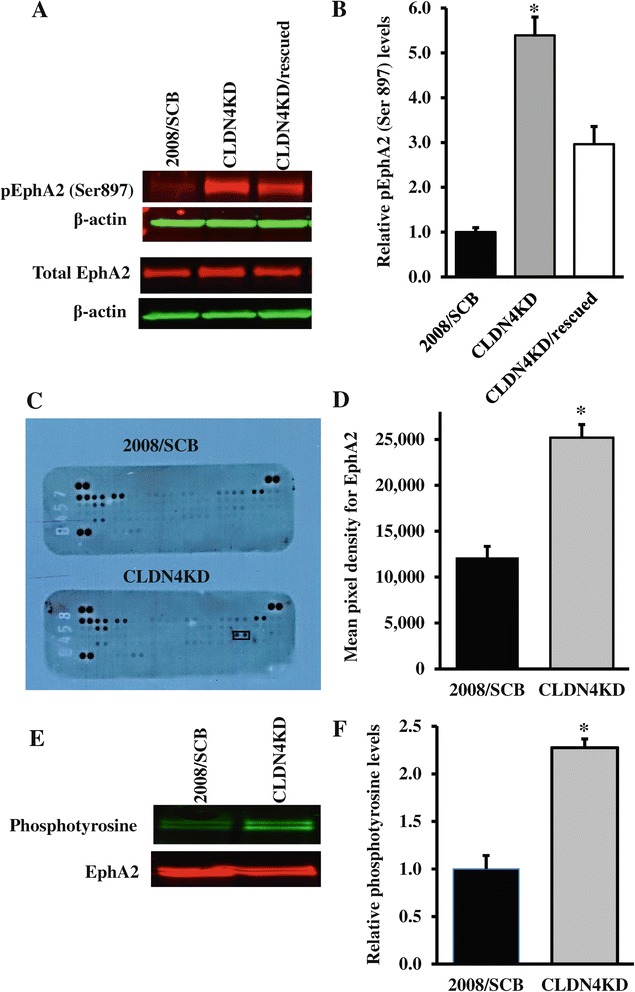
**CLDN4 knockdown affects the phosphorylation and kinase activity of EphA2. (A)** Representative Western blot showing the effect of CLDN4 knockdown and re-expression on EphA2 Ser897 phosphorylation. **(B)** Histogram showing the mean ± SEM level of pEphA2-Ser897 determined from 6 independent experiments expressed as the fold change relative to that in the scrambled siRNA control 2008/SCB cells after normalization to β-actin. ^*^ *p* < 0.05 versus 2008/SCB. **(C)** Measurement of EphA2 kinase activity in 2008/SCB and CLDN4KD cells using a human phospho-receptor tyrosine kinase array containing 49 different kinase substrates that include EphA1-5, EphA10 and EphB1-4, 6. **(D)** Quantification of the relative level of EphA2 tyrosine phosphorylation by densitometry. Results are presented as mean ± SEM (n = 3). ^*^ *p* < 0.05 versus 2008/SCB. **(E)** Detection of phosphotyrosine in immunoprecipitates prepared by using anti-EphA2 antibodies. Immunoprecipitates were probed with pan anti-phosphotyrosine antibody conjugated to horseradish peroxidase (HRP). **(F)** Histogram showing the average level of phosphotyrosine determined from 3 independent experiments expressed as the fold change relative to that in the scrambled siRNA control 2008/SCB cells after normalization to total EphA2. Results are presented as mean ± SEM (n = 3). ^*^ *p* < 0.05 versus 2008/SCB.

Phosphorylation of EphA2 on S897 is believed to mediate oncogenic signaling from this receptor. The extent of S897 phosphorylation was quantified by Western blot analysis using a phospho-S897-specific antibody. Figure 3A shows a representative Western blot, and Figure 3B shows the results of quantification of 3 independent Western blot analyses. The results indicate that the ratio of the steady-state level of S897 phosphorylation to total EphA2 was a 5.4 ± 0.2 times higher in the CLDN4KD cells than in the 2008/SCB cells (*p* < 0.05). When CLDN4 was re-expressed, the level of EphA2 phosphorylation on S897 in the CLDN4KD-rescued cells was reduced by 1.8 ± 0.5 -fold (*p* > 0.05) as compared with that in CLDN4KD cells. Thus, knockdown of CLDN4 was associated with a large increase in S897 phosphorylation and this was reversed by 37.5% when CLDN4 was re-expressed (*p* > 0.05).

To directly measure EphA2 activity we used a human phospho-receptor tyrosine kinase (RTK) array capable of simultaneously quantifying the tyrosine phosphorylation levels of 49 different RTKs. As shown in Figure 3C, the only RTK in this panel that demonstrated an increase in the phosphorylation was EphA2. Figure 3D presents the quantification and indicates that the phosphorylation of EphA2 was 2.1 ± 0.2 -fold higher in the CLDN4KD cells than in the 2008/SCB cells (*p* < 0.05). Thus, the 5.8 -fold increase in EphA2 phosphorylation on Ser897 was accompanied by a smaller but significant increase in its tyrosine phosphorylation. To confirm and extend the result of the RTK array analysis, we performed an immunoprecipitation study to detect the relative level of phosphotyrosine in EphA2 immunoprecipitates. As shown in Figure 3E and 3F, an increased level of tyrosine phosphorylation was also observed in EphA2-enriched precipitates prepared from CLDN4KD cells as compared with the control 2008/SCB cells.

### CLDN4 knockdown causes EphA2 mislocalization

**Figure 4:**
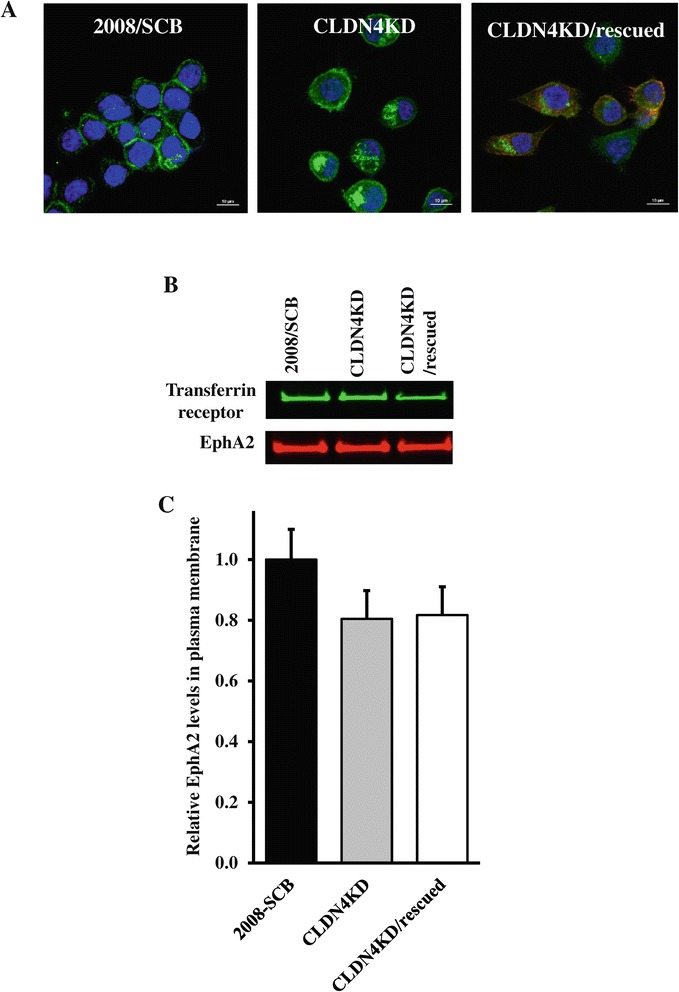
**Effect of CLDN4 knockdown on EphA intracellular distribution. (A)** The distribution of EphA2 in the 2008/SCB, CLDN4KD and CLDN4KD/rescued cells was visualized by immunofluorescent staining using anti-EphA2 antibody (green). Cell nuclei were counterstained with DAPI (blue). **(B)** Detection of EphA2 in biotinylated plasma membrane preparations by Western blot analysis using anti-EphA2 and anti-transferrin receptor antibody to provide a lane loading control. **(C)** Histogram summarizing the results of four independent Western blots.

In non-neoplastic epithelial cells EphA2 is reported to be localized to sites of cell-cell contact and this is dependent on the proper function of E-cadherin [21]. In the absence of functional E-cadherin, EphA2 was found to be redistributed into membrane ruffles and its localization could be restored by re-expressing E-cadherin. Interestingly, we previously found that E-cadherin expression was significantly reduced in the CLDN4KD cells compared with the 2008/SCB cells [18] and this encouraged exploration of EphA2 localization in the CLDN4KD cells. As shown in Figure 4A, and in Additional file 1: Figure S1 where co-staining with phalloidin was performed to identify the cell membrane, in the 2008/SCB cells EphA2 was located at the cell surface and concentrated at cell-cell contact sites. In the CLDN4KD cells, in addition to an overall increase in the amount of EphA2, a fraction of it was located in intracellular structures. Re-expression of CLDN4 in the knockdown cells partially restored the normal distribution of EphA2. Analysis of biotinylated cell surface proteins did not disclose a reduction in the amount of EphA2 in the plasma membrane in the CLDN4KD cells, or an increase in the CLDN4KD/rescued cells, suggesting that the excess EphA2 observed in the Western blot analysis was associated with the increased amount of EphA2 in an intracellular location.

### E-cadherin plays a minor role in modulating the effect of CLDN4 knockdown on EphA2

**Figure 5:**
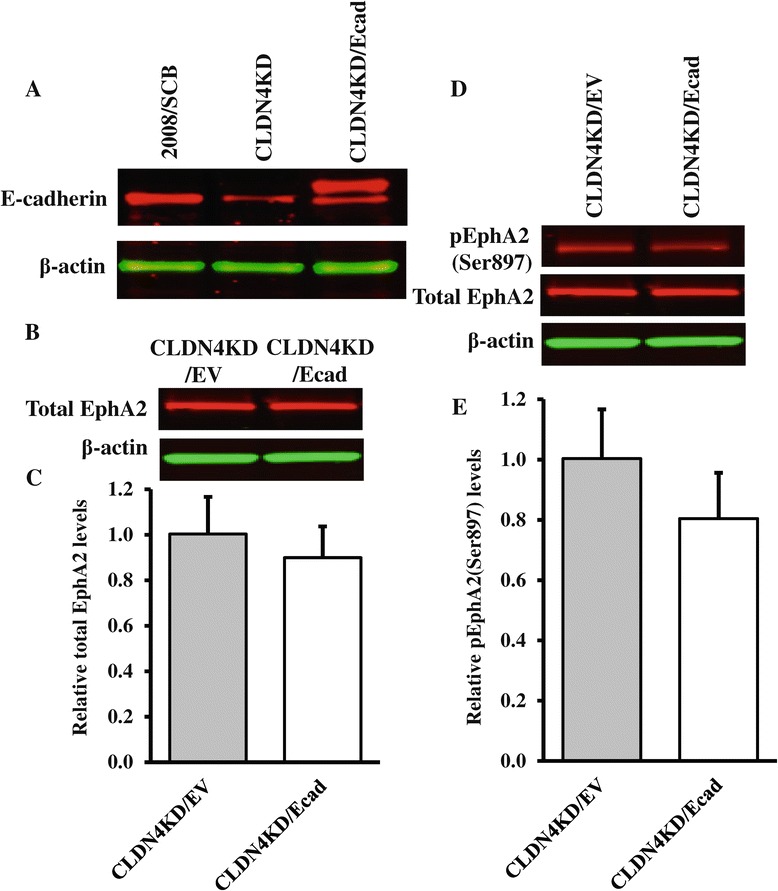
**Re-expression of E-cadherin in CLDN4KD cells does not significantly reduce total or pEphA2-Ser897. (A)** Western blot documenting increased expression of total E-cadherin in CLDN4KD/Ecad cells. **(B)** Representative Western blot showing total EphA2 levels in CLDN4KD/EV and CLDN4KD/Ecad cells. **(C)** The histogram shows the mean level of total EphA2 protein determined from 3 independent experiments expressed as the fold change relative to that in the empty-vector transfected control CLDN4KD/EV cells after normalization to β-actin. **(D)** Representative Western blot showing phosph-EphA2 levels in CLDN4KD/EV and CLDN4KD/Ecad cells. **(E)** The histogram shows the mean phospho-EphA2/total EphA2 ratio in CLDN4KD/EV and CLDN4KD/Ecad cells determined from 3 independent experiments. The data are expressed as mean ± SEM (n = 3).

To determine whether the effect of CLDN4 knockdown on the level of phosphorylation and localization of EphA2 was primarily due to the loss of CLDN4, or whether this was a secondary effect of the loss of E-cadherin that accompanies knockdown of CLDN4, E-cadherin was forcibly re-expressed in the CLDN4KD cells. Figure 5A documents the decrease in E-cadherin level when CLDN4 was knocked down, and its restoration when the CLDN4KD cells were engineered to re-express E-cadherin. As shown in Figures 5B-E, re-expression of E-cadherin did not result in significant changes in the levels of total EphA2 (Figure 5B and 5C) or in pEphA2(Ser897) (Figure 5D and 5E). These results indicate that E-cadherin was less effective than CLDN4 at restoring total EphA2 levels or shutting down the oncogenic signaling suggesting that the effect of CLDN4 knockdown on EphA2 was not primarily due to the associated loss of E-cadherin.

### Over-expression of CLDN4 decreases oncogenic EphA2 signaling even in the absence of E-cadherin

**Figure 6:**
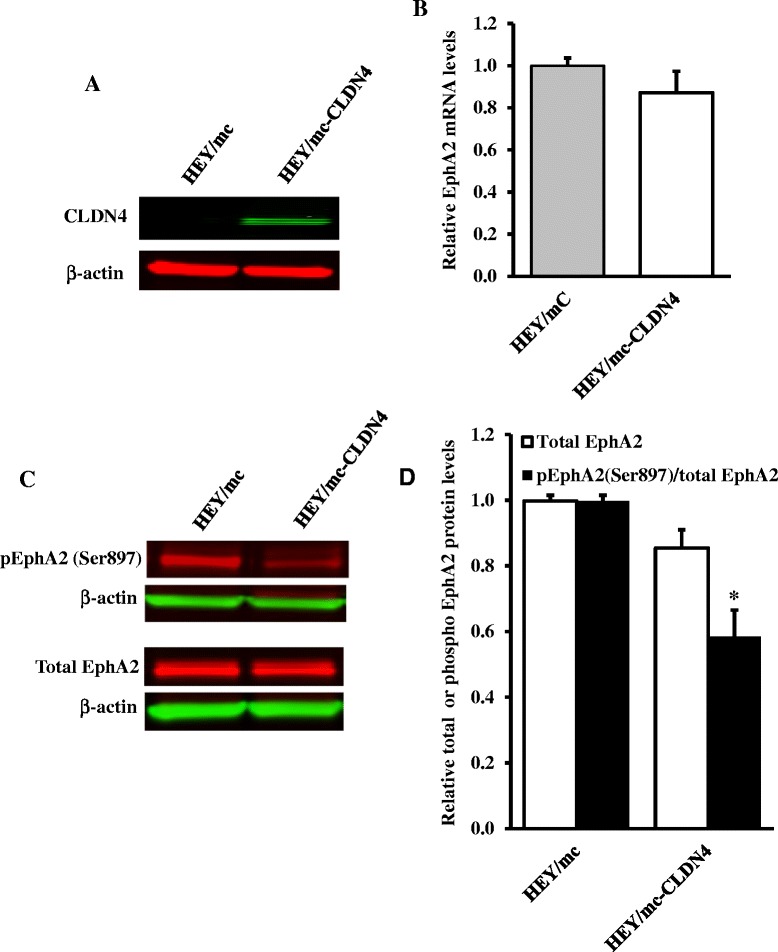
**Over-expression of CLDN4 diminishes EphA2 oncogenic signaling even in the absence of E-cadherin. (A)** Western blot analysis confirming increased expression of CLDN4 in HEY/mc-CLDN4 cells. **(B)** qRT-PCR analysis of the effect of CLDN4 over-expression on EphA2 mRNA levels. **(C)** Representative Western blot showing the effect of over-expression of CLDN4 in HEY cells on total EphA2 protein levels and EphA2 phosphorylation. **(D)** The histogram shows the mean level of the protein determined from 3 independent experiments. The relative total EphA2 levels in the HEY/mc-CLDN4 cells was expressed as the fold change relative to that in the HEY/mc cells after normalization to β-actin. The extent of EphA2 phosphorylation in the HEY/mc-CLDN4 cells was expressed as the ratio relative to that in the control HEY/mc cells after normalization to respective total EphA2 levels. Results presented are mean ± SEM (n = 3). ^*^ *p* < 0.05 versus HEY/mc.

**Figure 7:**
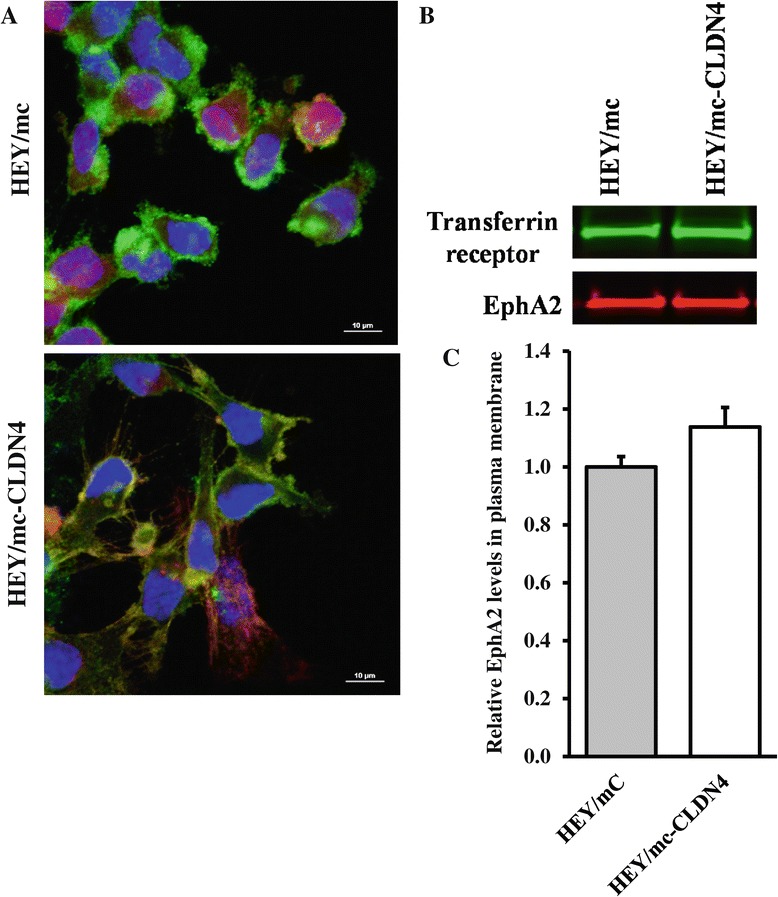
**Effect of CLDN4 on the subcellular localization of EphA2 in the absence of E-caderin. (A)** Upper panel, HEY/mc cells expressing mCherry (red) stained with anti-EphA2 antibody (green). Lower panel, HEY/mc-CLDN4 cells expressing mCherry fused to CLDN4 stained with anti-EphA2 antibody (green). Cell nuclei were counterstained with DAPI (blue). Yellow staining indicates co-localization. **(B)** EphA2 levels in the plasma membrane of HEY/mC and HEY/mc-CLDN4 cells as determined by Western blot analysis of biotinylated surface proteins. **(C)** Histogram summarizing the results of 3 independent Western blots. Data presented are mean ± SEM (n = 3).

To further isolate the regulation of EphA2 by CLDN4 from the effect of loss of CLDN4 on E-cadherin level, CLDN4 was over-expressed in the human ovarian carcinoma HEY cell line that does not express E-cadherin. Figure 6A documents the increase in CLDN4 expression in the HEY cells and Figures 6B and 6C show that this had no significant effect on EphA2 mRNA or total protein level. However, while CLDN4 expression did not significantly change the level of total EphA2 protein (*p* > 0.05), overexpression of CLDN4 did decrease the ratio of pEphA2(S897) to the total (*p* < 0.05) (Figure 6C and 6D). Figure 7A shows that over-expression of CLDN4 reduced the amount of intracellular EphA2 even in the absence of E-cadherin expression but as in the 2008/SCR cells this was not associated with a change in the total plasma membrane EphA2 (Figure 7B and 7C). Thus, CLDN4 can function independently of E-cadherin to regulate EphA2 to suppress its oncogenic signaling and alter its cellular localization. This is consistent with our prior studies in the HEY cell line showing that increased CLDN4 suppressed both migration and invasion [18].

### CLDN4 controls the switch to AKT-EphA2 pro-oncogenic signaling via β-catenin

**Figure 8:**
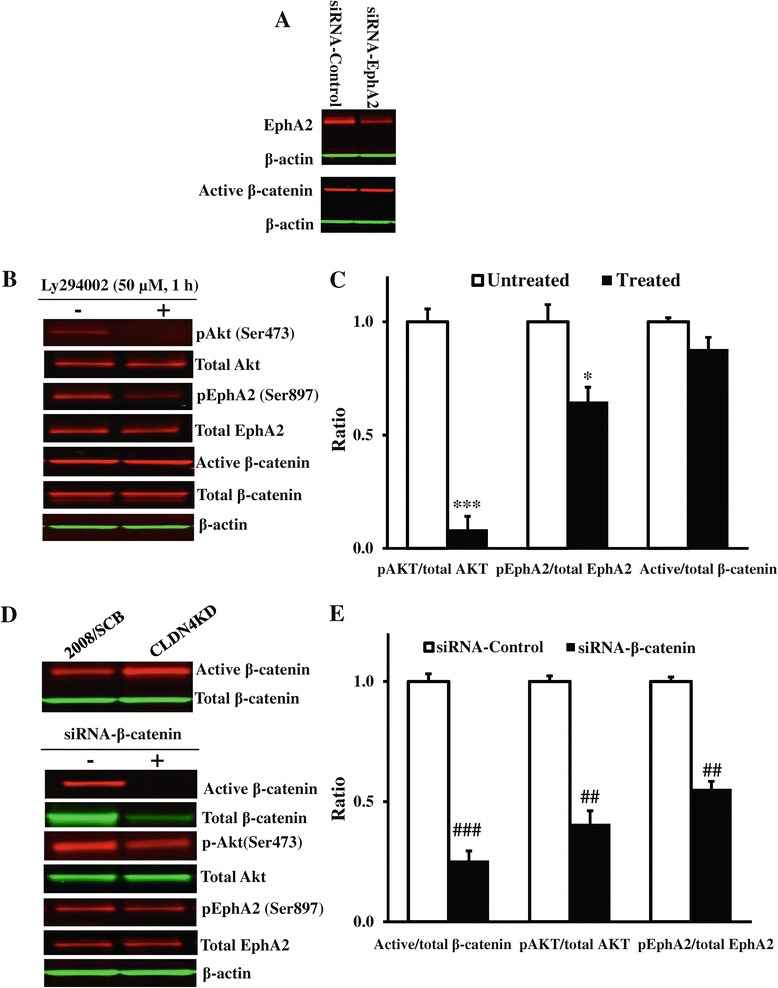
**Analysis of the pathway by which loss of CLDN4 activates EphA2. (A)** Western blot analysis showing no changes of active β-catenin levels when EphA2 was knocked down in the CLDN4KD cells. **(B)** Representative Western blot showing the level of pAkt(Ser473), pEphA2(Ser897) and dephospho-β-catenin in the CLDN4KD cells untreated or treated with 50 μM PI3K inhibitor LY294002 for 1 h. **(C)** The histogram showing the mean level of pAkt(Ser473), pEphA2(Ser897) and dephospho-β-catenin expressed as the ratio relative to that in untreated CLDN4KD cells after normalization for their respective total protein. Results presented are mean ± SEM (n = 3). ^***^ *p* < 0.001, ^*^ *p* < 0.05 versus untreated CLDN4KD. **(D)** Representative Western blot showing that active β-catenin was increased in the CLDN4KD cells (top panel) and siRNA-mediated knockdown of β-catenin reduced the levels of pAkt(Ser473) and pEphA2(Ser897) in the CLDN4KD cells (bottom panel). **(E)** Histogram showing the mean levels of pAkt(Ser473) and pEphA2(Ser897) determined from 3 independent experiments expressed as the fold change relative to that in the scrambled siRNA transfected CLDN4KD cells after normalization to β-actin. Results are presented as mean ± SEM (n = 3). ^###^ *p* < 0.01, ^##^ *p* < 0.01 versus scrambled siRNA transfected CLDN4KD.

The loss of CLDN4 in 2008 cells is associated with down-regulation of E-cadherin, a substantial increase in β-catenin signaling and the level of the active dephosphorylated form of β-catenin in the nucleus [18]. Our prior work has also demonstrated that loss of CLDN4 resulted in activation of the PI3K pathway as evidenced by increased AKT phosphorylation, elevated cellular PIP3 content and PI3K activity [22]. Thus, one route by which loss of CLDN4 activates EphA2 may be through the sequential activation of PI3K and AKT by β-catenin. Alternatively, loss of CLDN4 may activate PI3K leading to the phosphorylation of AKT and EphA2 and the subsequent activation of β-catenin secondary to the oncogenic signaling from EphA2. To distinguish between these alternatives, we transiently knocked down EphA2 and looked for an effect on the dephosphorylated active form of β-catenin to determine whether β-catenin was upstream or downstream of EphA2. As shown in Figure 8A, there was a clear reduction in EphA2 level but no effect on the level of active β-catenin in the CLDN4KD cells. Next, the activity of PI3K was inhibited by exposure of the cells to the PI3K inhibitor LY294002. This drug reduced the level of pAKT(S473) dramatically and to a lesser extent of pEphA2(S897) but had no detectable effect on the level of the active form of β-catenin (Figures 8B and 8C). Both of these results suggested that the activation of PI3K and AKT was being driven primarily by upstream β-catenin. To confirm this, we transiently knocked down β-catenin in the CLDN4 knockdown cells. As shown in Figure 8D, the siRNA was very effective in reducing the level of dephospho-β-catenin, and this resulted in a significant decrease of pEphA2(S897) (Figure 8D and 8E). Thus, the data is most consistent with the concept that loss of CLDN4 results in enhanced β-catenin and activation of PI3K and AKT upstream of EphA2 independent of effects mediated by loss of E-cadherin.

## Discussion

CLDN4 plays an important role in tight junction formation and paracellular permeability in epithelial monolayers. We previously discovered that CLDN4 has quite marked effects on tumor growth rate and metastatic potential in the human cervical carcinoma cell line 2008. Knockdown of CLDN4 increased migration, invasion and *in vivo* growth rate and metastatic colonization of the lungs, and these effects were shown to be specific to the loss of CLDN4 as re-expression of CLDN4 rescued the phenotype [18]. Forced expression of CLDN4 in the HEY cell line that does not endogenously express this protein produced similar effects. A search for the mechanism by which CLDN4 produces such pervasive effects led us to consider EphA2.

Increased expression of EphA2 is found in many types of cancer and is associated with aggressive features [23]. The phosphorylation of a single serine residue in EphA2, S897, by AKT switches on ligand-independent promotion of cell migration and invasion. The switch is turned off when the site becomes dephosphorylated upon the binding of EphA2 to its normal ligand ephrin-A1 [11]. To determine how CLDN4 might affect EphA2 we examined changes in EphA2 expression, location and activity when CLDN4 was constitutively knocked down and found that knockdown of CLDN4 produced an increase in total EphA2 expression at both transcriptional and translational level. Knockdown of CLDN4 also resulted in enhanced phosphorylation at S897 and an increase in tyrosine phosphorylation detected using a human phospho-RTK array. It was also accompanied by a greater amount of intracellular EphA2 in the absence of a clear change in total plasma membrane EphA2, a change whose significance is uncertain. All of these effects were specific as they are variably rescued by re-expression of CLDN4, and this effect of CLDN4 was also observed when it was over-expressed in the HEY cell line that does not normally express this protein. It should be recognized that although EphA2 is reported to be a direct transcriptional target of Raf-MARK pathways [24,25], the exact contribution of this protein and genetic regulation of its expression to the specific processes involved to tumor formation, maintenance, and progression is extremely complex and dependent on many factors. The molecular basis of how loss of CLDN4 regulates EphA2 gene expression remains to be determined in the future studies.

E-cadherin plays a central role in maintaining the integrity of epithelia and the polarity of epithelial cells. Loss of E-cadherin expression or function is common in cancer cells and is associated with disruption of cell-cell contacts and increased aggressive behavior and metastasis [26]. E-cadherin is also an important regulator of EphA2. E-cadherin is required for the localization of EphA2 at cell-cell contacts and in the absence of E-cadherin EphA2 is redistributed into membrane ruffles where it cannot engage with membrane-bound ligand ephrin-A1 on adjacent cells [21,26,27]. We previously noted that knockdown of CLDN4 is accompanied by reduced E-cadherin expression at both mRNA and protein levels [18], and this led us to investigate whether simply re-expressing E-cadherin could restore normal EphA2 localization to the sites of cell-cell contact and reduce its pro-oncogenic signaling. However, re-expression of E-cadherin did not clearly increase the fraction of EphA2 found in the plasma membrane, and it did not significantly reduce either total EphA2 levels or the pEphA2(S897) level. Thus, restoring the expression of E-cadherin by itself did not turn off the oncogenic switch indicating the presence of an E-cadherin-independent mechanism of EphA2 regulation by CLDN4. Further evidence of such regulation by CLDN4 was provided by the observation that expression of CLDN4 in the HEY cells that do not express E-cadherin enhanced the localization of EphA2 to the plasma membrane, and reduced oncogenic signaling as evidenced by a significant reduction in pEphA2(S897).

The normal positioning of CLDN4 at the tight junction is known to limit activation of PI3K and AKT [22] and we considered the possibility that in the absence of oncogenic signaling activated by phosphorylation of EphA2 on S897 the activity of β-catenin would be limited. However, neither knockdown of EphA2 nor inhibition of PI3K with LY294002 reduced the active form of β-catenin suggesting that the activation of PI3K, AKT and EphA2 was a consequence rather than the cause of the increase in dephospho-β-catenin. This was confirmed when knockdown of β-catenin was shown to reduce the level of pEphA(S897) in the CLDN4 knockdown cells. At this point the details of how CLDN4 limits β-catenin activity remain to be worked out. However, it is of interest that EphA2 can phosphorylate the cytoplasmic tail of CLDN4 which decreases its integration into tight junction and favors more malignant behavior [28]. This suggests the possibility of an amplification loop such that small changes in the level of pEphA2(S897) may be augmented by the subsequent loss of CLDN4 from the tight junction that results in further EphA2 phosphorylation.

## Conclusions

Taken together, our results suggest that CLDN4 serves to restrain the pro-oncogenic signaling of EphA2 by limiting the activity of β-catenin and PI3K and preventing phosphorylation of EphA2 on S897 by AKT. While it should be recognized that therapeutic approach involving enhancement of tight junction may represent a technical challenge, this observation suggests that interventions directed at enhancing the level or functional activity of CLDN4 may be still of therapeutic interest.

## Methods

### Cells and cell culture

Human cervical carcinoma 2008 cells and human ovarian carcinoma HEY cells were grown in RPMI 1640 medium supplemented with 10% fetal bovine serum, 100 U/ml penicillin, and 100 mg/ml streptomycin. For many years 2008 was believed to be an ovarian cancer cell line, but recent genetic test has shown it to be identical to the ME-180 cervical carcinoma cell line [29], and testing by ATCC of cells submitted from this laboratory has independently confirmed this result. The 2008 sublines of 2008-CLDN4KD-5.5 and CLDN4KD-4 rsc, identified here as CLDN4KD and CLDN4KD/rescued, in which CLDN4 was knocked down [30] and the knockdown was rescued by reintroduction of an siRNA-resistant CLDN4 construct into the CLDN4KD cells [31], and the CLDN4-expressing subline of HEY (HEY/mc-CLDN4) plus the empty vector-transfected control cells (HEY/mc) [22], were cultured in the same medium as the wild type 2008 with the addition of 10 *μ*g/ml puromycin. The GFP-E-cadherin expressing subline of CLDN4KD (CLDN4KD/Ecad), and the empty vector-transfected control (CLDN4KD/EV) [22] were grown in the same medium with the addition of 400 μg/ml G418.

### Quantitative real time PCR

RNA was extracted with TRIzol reagent (Invitrogen, Carlsbad, CA). First-strand cDNA was synthesized using SuperScript II reverse transcriptase (Invitrogen) and random primers. Quantitative real-time PCR was performed using the Bio-Rad iCycler iQ detection system in the presence of SYBR Green I dye (Bio-Rad Laboratories, Inc, Hercules, CA). β-actin was used as reference gene and relative mRNA levels were determined using the 2^(−ΔΔCt)^ method. A 1-unit difference of *C*_t_ value represents a two-fold difference in the level of mRNA.

### Western blot analysis

Whole-cell lysates were prepared in RIPA lysis buffer (Sigma-Aldrich, St. Louis, MO) with Halt Protease and Phosphatase Inhibitor Cocktails (Thermo Scientific, Waltham, MA) and centrifuged at 12,000 × g for 10 min at 4°C. The supernatant was loaded on SDS-PAGE and separated by electrophoresis. A Bio-Rad Trans-Blot system was used to transfer the proteins to Immobilon-P FL membranes (Millipore, Bedford, MA). Membranes were blocked for 1 h at room temperature in Odyssey Blocking Buffer (Li-Cor Biosciences, Lincoln, NE), followed by incubation overnight at 4°C with specific antibodies. The following primary antibodies were used: T-EphA2 (Cell Signaling Technology, Danvers, MA), p-EphA2(Ser897) (Cell Signaling Technology), p-EphA2(594) (Cell Signaling Technology), actin (Sigma-Aldrich). After washing 3 times for 5 min each at room temperature in TRIS buffered saline containing 0.1% Tween-20, the blots were incubated for 1 h at room temperature with fluorescently labeled secondary antibody (Li-Cor Biosciences) diluted 1:10,000 in the Odyssey Blocking Buffer containing 0.1% Tween 20 and 0.02% SDS. After 3 washes for 5 min each in TRIS buffered saline containing 0.1% Tween-20 and rinse with PBS, the blots probed with fluorescently labeled antibody were imaged using an Odyssey Infrared Imager (Li-Cor Biosciences). The ratio of protein levels in both experimental cell types was calculated densitometrically after normalization to the level of β-actin and expressed as the fold change relative to the ratio in control cells.

### EphA2 immunoprecipitation (IP)

Whole-cell lysates were diluted into IP buffer (50 mM phosphate, pH 7.2, 200 mM NaCl, 2.5 mM dithiothreitol, and 0.5% n-dodecyl-β-d-maltoside) after which they were precleared with protein A/G plus agarose beads (Thermo Scientific; Rockford, IL) and then centrifuged at 1200 × g for 5 min. Anti-EphA2 antibody was added to the precleared supernatant at a dilution of 1:100 and rotated at 4°C for 60 min after which 10 μL of protein A/G plus agarose beads were added and the mixture was rotated overnight at 4°C. The beads were then washed 5 times in IP buffer and bound proteins were eluted at 37°C with 2× sample buffer (125 mM Tris, pH 6.8, 2 mM EDTA, 6% SDS, 20% glycerol, 0.25% bromophenol blue and 5% β-mercaptoethanol). The eluates were assessed by Western blot analysis using pan anti-phosphotyrosine antibody (R & D Systems, Minneapolis, MN) and anti-EphA2 antibody (Cell Signaling Technology).

### Biotinylation of plasma membrane proteins

Assay of cell surface levels of EphA2 in the 2008/SCB, CLDN4KD, HEY/mc and HEY/mc-CLDN4 was performed using the Cell Surface Protein Isolation kit (Thermo Scientific; Rockford, IL). Cells grown to 80–90% confluence in a 145 mm plate were biotinylated, lysed and cell surface proteins isolated and eluted according to the manufacturer's instructions. The eluted proteins were subjected to Western blot analysis with anti-EphA2 antibody with anti-transferrin receptor used as a loading control.

### Immunofluorescent microscopy

Cells were grown on polylysine-coated eight-well chamber slides, fixed in PBS containing 4% formaldehyde at 37°C for 20 min, followed by permeabilization in PBS- 0.3% Triton X-100 for 10 min at room temperature. After 3 washes with PBS, the gels were blocked for 1 h at room temperature with 3% goat serum in PBS followed by incubation overnight at 4°C with primary antibody T-EphA2 diluted to 1:200 in the antibody dilution buffer (PBS-3% goat serum-0.3% Triton X-100). After washing 3 times for 5 min each at room temperature in PBS, the cells were incubated for 1 h at room temperature with Alexa Fluor® 488 dye (Life Technologies, Carlsbad, CA) diluted to 1:1000 in antibody dilution buffer, followed by 3 additional washes following which the cells were mounted with Gelvatol mounting medium (Fisher Scientific, Pittsburgh, PA). Images were captured using a Nikon A1R Confocal STORM super-resolution system.

### Would healing/Scratch assay

Cells were grown to confluence on 12-well cell culture plate and scratch was made through the cell monolayer using a pipette tip. After washing with Hank’s balanced salt solution, fresh culture medium was added and the cells were incubated at 37°C in a humid environment with 5% CO_2_. Wound closure was observed and photographed every 15 min after making the scratch to monitor the invasion of cells into the wounded area. Slidebook (v5.0) software was used to track cell trajectories by nuclear position over time of 16 – 21 cells in 4 separate microscopic fields in each group, and average migration speed was calculated. The experiment was performed twice and assayed in triplicate.

### Statistical analysis

All data are presented as mean ± SEM of a minimum of 3 independent experiments. Significance of differences between groups was determined using the Student *t* test or the Dukkett one-way analysis of variance (ANOVA).

## Additional file

Additional file 1: Figure S1. Knockdown of CLDN4 alters EphA intracellular distribution. The distribution of EphA2 (green) in the 2008/SCB, CLDN4KD and CLDN4KD/rescued cells was visualized by immunofluorescent staining using anti-EphA2 antibody. Phalloidin (purple) was used to stain actin. Red fluorescence is from the mCherry-CDLN4.
