# Supplementary material for: Claudin-4 controls the receptor tyrosine kinase EphA2 pro-oncogenic switch through β-catenin
Source: Cell Commun Signal. 2014 Oct 25;12:59. doi: 10.1186/s12964-014-0059-5 (PMC4212103; doi:10.1186/s12964-014-0059-5)
Supplement: Additional file 1: Figure S1. — Knockdown of CLDN4 alters EphA intracellular distribution. The distribution of EphA2 (green) in the 2008/SCB, CLDN4KD and CLDN4KD/rescued cells was visualized by immunofluorescent staining using anti-EphA2 antibody. Phalloidin (purple) was used to stain actin. Red fluorescence is from the mCherry-CDLN4. [file 12964_2014_59_MOESM1_ESM.pptx]

## Slide 1
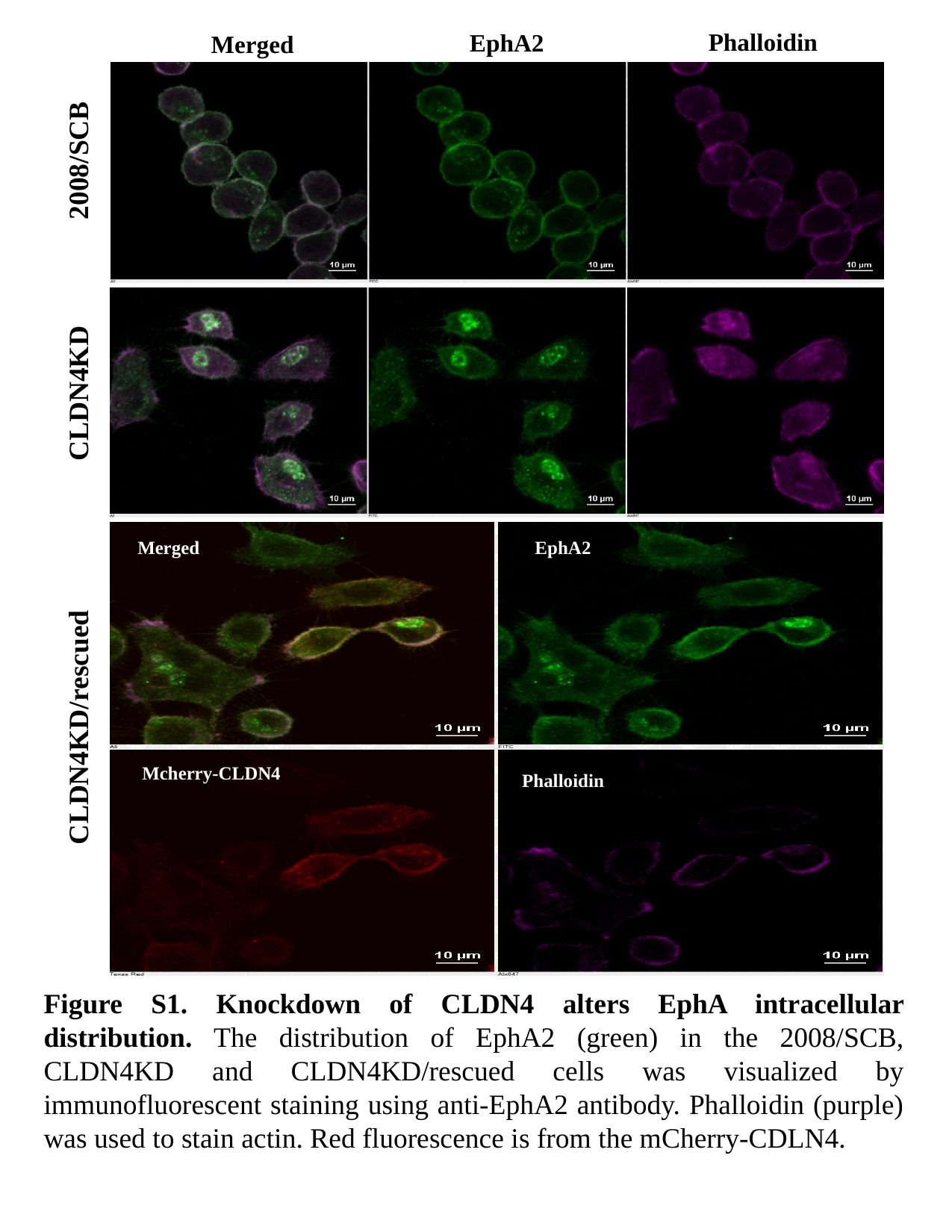

Phalloidin
EphA2
Merged
 CLDN4KD/rescued CLDN4KD 2008/SCB
Merged
EphA2
Mcherry-CLDN4
Phalloidin
Figure S1. Knockdown of CLDN4 alters EphA intracellular distribution. The distribution of EphA2 (green) in the 2008/SCB, CLDN4KD and CLDN4KD/rescued cells was visualized by immunofluorescent staining using anti-EphA2 antibody. Phalloidin (purple) was used to stain actin. Red fluorescence is from the mCherry-CDLN4.
